# 

**Published:** 1802-08-01

**Authors:** 


### To CORRESPONDENTS.

*Communications are received from Drs. Ryan and Noehden; and several anonymous Correspondents on Quackery, whose address we request.——We apprehend Mr. Marson has been anticipated by Mr. Lownds, of St. Paul's Church Yard.—— We inform Pyrrho, jun. that Kate Hudson had long been in the habit of sleeping with pins, &c. in her mouth.*

---

### ERRATUM.

Page 52, line 3, for *Bucks*, read *Berks.*

---

THE LONDON MEDICAL REVIEW *having been discontinued for want of due Encouragement, the Readers of that Work and the Public at large are informed, that henceforward all new Books, connected with Medicine and Natural Philosophy, will be regularly analyzed in the Medical and Physical Journal. This Work, by its punctual Analysis of all new Books as fast as they appear, will consequently recommend itself to the Readers of the London Medical Review, and be increased in Value to its own immediate Subscribers.*

